# Laminated Glass Plates Subjected to High-Velocity Projectile Impact and Their Residual Post-Impact Performance

**DOI:** 10.3390/ma15238342

**Published:** 2022-11-23

**Authors:** Petr Konrád, Petr Hála, Jaroslav Schmidt, Alena Zemanová, Radoslav Sovják

**Affiliations:** 1 Experimental Centre, Faculty of Civil Engineering, Czech Technical University in Prague, Thákurova 7, 166 29 Prague, Czech Republic; 2 Department of Mechanics, Faculty of Civil Engineering, Czech Technical University in Prague, Thákurova 7, 166 29 Prague, Czech Republic; 3 Department of Geotechnics, Faculty of Civil Engineering, Czech Technical University in Prague, Thákurova 7, 166 29 Prague, Czech Republic

**Keywords:** glass, laminated, ballistic, impact, 9 mm, residual, analytical, simulations, bending

## Abstract

This study aims to analyze the performance of laminated glass against ballistic loading and investigates its residual load-bearing capacity. Two groups of specimens were used in quasi-static four-point bending experiments, first without prior ballistic damage and then with it. The main objective was to compare the load-bearing capacity of these two groups to see the effect of ballistic damage. Three different layer compositions were used. The ballistic loading was conducted using an in-service 9 mm bullet fired from a semiautomatic carbine with the glass specimens hanging on steel ropes in a free boundary setup. Numerical simulation and analytical methods were used and validated against the measured response of the undamaged specimens. The simulations were in good agreement with the experimental results. All of the glass specimens were able to withstand the ballistic loading, and the subsequent performance during the quasi-static bending loading was similar to that of the undamaged specimens. The quality of the glass edges seemed to be more important than ballistic damage. The front-plate damage played a negligible role, and the back-plate damage needed to be extensive to influence subsequent performance. Provided that ballistic damage is mainly localized only to the centers of the plates, it did not affect the post-impact loading capacity.

## 1. Introduction

Glass is a brittle material that offers poor resistance against impact loading and no post-fracture loading capacity. This characteristic can be negated by pairing glass with polymer interlayers to form laminated elements. Such an element then offers ductile post-cracking behavior, as the interlayer is firmly bonded to the glass ply even when cracked or shattered. During loading, the glass plies do not suddenly fail all at once, but gradually, contributing to the ductile behavior. Laminated glass is used in many applications in the construction industry to form non-structural decorations and structural load-bearing elements. However, the brittle nature of the glass plies is still a concern, especially when we take into consideration the relatively unlikely, yet high-risk, loading scenarios such as projectile impacts and blast-accelerated debris impacts.

In recent years, numerous studies have investigated the impact performance of laminated glass, mostly focusing on the low-velocity impacts and numerical modeling of the resulting behavior [1,2,3,4,5]. Another field where various glass impacts are studied is vehicle manufacturing [6,7]. Zhang et al., for example, studied different interlayer materials when subjected to impact loading [8]. Temperature effects were analyzed in a study conducted by Wu et al. [9]. There were also studies dealing with specific anti-ballistic designs and advances in that field [10,11]. However, ordinary structural elements produced with standard laminated glass can also be subjected to extraordinary loads. In such cases, the actual ballistic performance does not need to be the top priority (stopping the projectile, limiting the amount of secondary debris), unlike the residual post-impact load-bearing capacity. In other words, the elements should still fulfill their structural roles even when damaged, so as to not cause further progressive collapse with more serious consequences. It is then necessary to identify the post-impact capacity to be able to design structures with this kind of loading in mind.

Post- and pre-impact capacities are evaluated using quasi-static experiments. Bending tests are the most common. Biolzi and Simoncelli [12] used a 3-point configuration with large plates (2 glass layers with 1 interlayer) to also investigate the bending performance when the top compressed layer was pre-cracked. They found that, in this case, the plate performed similarly to the undamaged specimens. However, they indicated the importance of boundary conditions. Zhao et al. [13] also tested pre-cracked specimens using a square plate supported in its corners, where the effect of these specific boundary conditions was apparent. They concluded that the damage pattern prior to quasi-static testing plays a key role. Numerous other bending experiments can be found in the literature [14,15,16].

To subject a specimen to high-velocity impact loading, projectiles need to be accelerated by either compressed air or explosions. Ballistic testing is also used, employing common in-service ammunition. Projectile sizes, weights, and materials, and the means of securing tested specimens all differ. Mohagheghian et al. [17] used a steel frame in which they secured a laminated glass specimen impacted by a soft projectile, simulating a bird strike on a flying airplane. Deng et al. [18] also used a metal frame with a protective rubber gasket to simulate a train window subjected to impacts of small sand-blown grains of sand. Osnes et al. [19] ballistically tested laminated glass specimens that were also supported by a rigid metal frame with rubber pads. The projectile was a 7.62 mm armor piercing bullet.

In the authors’ previous studies, low-velocity impact experiments were conducted on specimens held only by steel ropes without any frames or other rigid supports [20,21]. This allowed for the more accurate collection of experimental data (accelerations, forces) for subsequent numerical simulations. Not using rigid supports also allows for the easier repeatability of the experiments. The influence of supports in dynamic loading is difficult to distinguish from material performance, so the elimination of supports is an efficient solution. The steel ropes did not influence the measurements, and the approach was overall suited for this type of analysis. Therefore, in this study, the same principle is used for high-velocity testing.

## 2. Materials and Methods

### 2.1. Specimen Description

**Figure 1:**
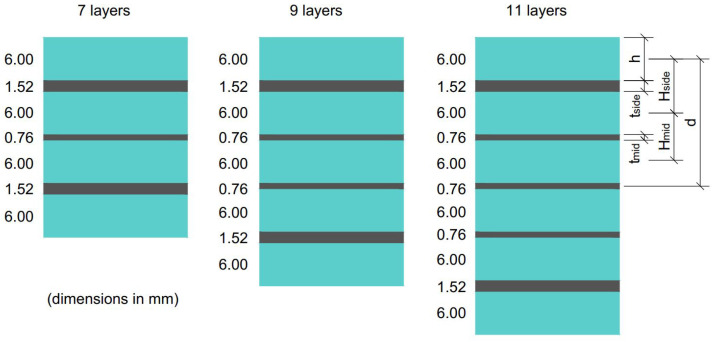
Glass and polymer interlayer configurations. Nominal thicknesses.

The laminated glass plates chosen for the experimental campaign had nominal dimensions of 1100 mm × 360 mm. Three different layer configurations were used: 7, 9, and 11 total layers. The detailed layer information is presented in Figure 1. Glass layers were produced with ordinary float soda–lime–silica glass, and polymer interlayers were composed of polyvinyl butyral (PVB).

### 2.2. Experimental Testing of Undamaged Specimens

**Figure 2:**
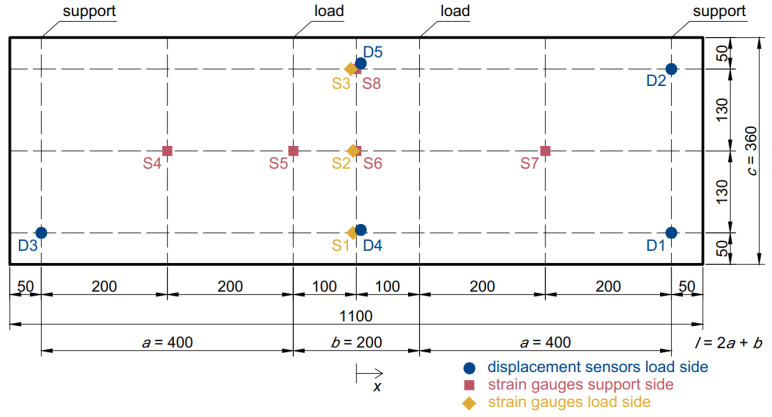
Setup of the four-point bending experiment.

The first specimen group was subjected to quasi-static four-point bending experiments. The configuration of the test corresponded to the standard setup [22] for the four-point bending test and is shown in Figure 2. The span between the supports was 1000 mm, and the distance between the loading points was 200 mm. The glass specimens were protected from the loading and support steel cylinders with rubber pads to prevent unwanted damage. Figure 2 also shows the positions of measuring equipment-linear variable differential transformer displacement sensors and glued strain gauges. The displacement sensors above the supports were used to offset the influence of the rubber pads from other displacement measurements. The experiment was displacement-controlled with a loading rate of 1 mm/min.

### 2.3. Analytical Method

The enhanced effective thickness (EET) analytical method was applied to calculate the displacement and strains. The method is explained in [23]. In the same study, the authors concluded that the EET method provides more accurate results compared to other simplified analytical methods, even for multilayered laminated glass. This method was chosen for the presented study because it represents a practical tool for the design of laminated glass that, on the one hand, is relatively simple, and provides, on the other hand, a relatively accurate prediction of the response. However, it needed to be adapted to a four-point bending configuration. First, the shape function g(x) needed to be identified on the basis of loading and boundary conditions. For the general case of a four-point bending, it is given as follows:

(1) g(x)=−F16(x+b2)3+12x2a+16a3−18l2afor−l/2≤x≤−b/2−F12x2a+16a3−18l2afor−b/2<x≤b/2−F16(x−b2)3−12x2a−16a3+18l2afor−b/2<x≤l/2

where *F* is half of the loading force (force acting on one of the two loading rollers), *a* is the distance between a support and the closest loading point, *b* is the distance between loading points, and *l* is the span between supports. The equations were written assuming that the origin of the system was in the center of the specimen (Figure 2). The next step was to calculate coefficient ψ using equation

(2) $$\psi =\frac{\int_{-l/2}^{l/2}{\left[g^{\prime \prime }\left(x\right)\right]}^{2}\;dx}{\int_{-l/2}^{l/2}{\left[g^{\prime }\left(x\right)\right]}^{2}\;dx}$$

which yields the following expression

(3) $$\psi =\frac{40a+60b}{16a^{3}+40a^{2}b+30ab^{2}+5b^{3}}$$

For the test configuration, the coefficient was equal to 9.915 m${}^{-2}$. For this geometry, using a coefficient equation for three-point loading (tabulated in [23]) would yield very similar value of 10 m${}^{-2}$.

Next, coefficient η reducing the thickness of the specimen could be calculated as

(4) $$\frac{1}{\eta }=1+\frac{E}{GcI_{tot}\left(\frac{H_{side}^{2}}{t_{side}}+\left(n-3\right)\frac{H_{mid}^{2}}{t_{mid}}\right)}\psi nIA\sum_{i=1}^{n}d_{i}^{2}$$

where *E* is the modulus of elasticity of glass (70 GPa), *G* is the shear modulus of the interlayer (0.4 MPa, see Section 2.4), *c* is the width of the specimen, $I_{tot}$ is the moment of inertia as if the specimen was monolithic, $H_{side}$ and $H_{mid}$ are the distances between centers of the neighboring glass plies on the sides of the specimen and in the middle, respectively, $t_{side}$ and $t_{mid}$ are the interlayer thicknesses of the outermost layer and the rest, respectively, *n* is the number of glass layers, *I* is the moment of inertia of one glass ply, *A* is the cross-sectional area of one glass ply, and *d* is the distance of the center of the glass ply to the center of the whole specimen (see Figure 1). This equation only applies to this specific case where the glass layers are identical, and only the outermost interlayers have different thicknesses than the rest. In the final step, the effective thicknesses of the whole specimen could be calculated. First, deflection-effective thickness ${\hat{h}}_{w}$ for the displacement calculations could be determined from the following equation

(5) $$\frac{1}{{\hat{h}}_{w}^{3}}=\frac{\eta }{nh^{3}+12h\sum_{i=1}^{n}d_{i}^{2}}+\frac{\left(1-\eta \right)}{nh^{3}}$$

where *h* is the glass ply thickness. Second, stress-effective thickness ${\hat{h}}_{i;\sigma }$ for calculating stress and strain is given by

(6) $$\frac{1}{{\hat{h}}_{i;\sigma }^{2}}=\frac{2\eta d_{i}}{nh^{3}+12h\sum_{i=1}^{n}d_{i}^{2}}+\frac{h}{{\hat{h}}_{w}^{3}}$$

where $d_{i}$ is the distance of center of the glass ply where the extreme stress is evaluated to the center of the whole cross-section.

### 2.4. Numerical Simulations

**Figure 3:**
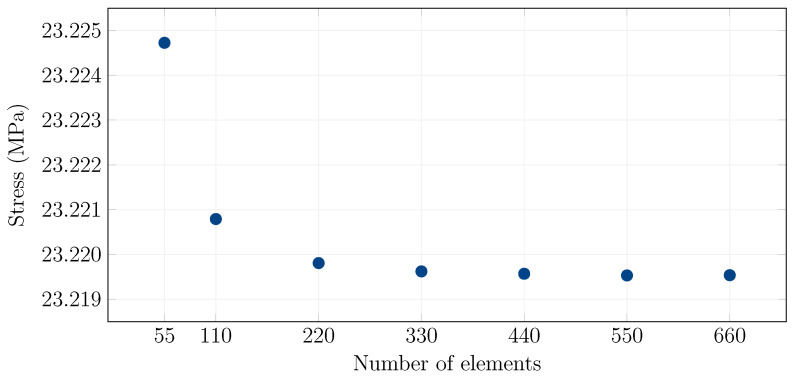
An example of a convergence test for the 7 layer configuration, bottom surface stress, center of the span.

The four-point bending experimental data of the undamaged specimens were further compared with finite-element simulations. Since the plates were supported and loaded equally along the entire width of the specimen, a simple Mindlin beam model was used for validation. Only half of the specimen was simulated using a symmetry condition. Each layer was discretized with 550 equidistantly distributed linear elements, and individual layers were bound by two assumptions: (i) glass and foil are incompressible across the thickness, (ii) the displacements of neighboring layers on the interfaces between layers are continuous without considering delamination. Such a number of elements is sufficient for the simulation, which is illustrated with a convergence diagram in Figure 3, where bottom tensile stress is plotted against the number of elements at approximately half of the simulation time. The glass was modeled as a purely elastic material with E=70 GPa and Poisson’s ratio ν=0.22, while the empirical rule at t/2 was used to take into account the viscoelastic behavior of the polymer interlayer. This rule supposes that the employed PVB shear modulus $G_{\mathrm{num}}$ in time *t* and at temperature *T* was evaluated from the relaxation shear function as

(7) $$\begin{array}{c} G_{\mathrm{num}}\left(t,T\right)=G_{\mathrm{viscoel}}\left(t/2,T\right)=G_{\infty }+\sum_{p=1}^{P}G_{p}\exp\left(-\frac{t/2}{a_{T}\left(T\right)\theta_{p,0}}\right). \end{array}$$

The parameters of the generalized Maxwell model in terms of Prony series $G_{\infty }$, $G_{p}$, and $\theta_{p,0}$ were fitted from shear relaxation experiments (Table 1). The temperature shift was taken into account using the William–Landel–Ferry equation

(8) $$\log_{10}a_{T}=-\frac{C_{1}\left(T-T_{0}\right)}{C_{2}+T-T_{0}},$$

with the actual temperature during the test of T=28°C, reference temperature of $T_{0}$, and shift parameters of $C_{1}$ and $C_{2}$ (Table 1). Details regarding the used viscoelastic model, calibration process, and experiments can be found in [24]. The value of G=0.4 MPa for the analytical solution was taken from the numerical simulation’s output at the time step when certain forces were reached. These forces were the averages of peak forces obtained from the experiments for one layer group, as reported in Section 3.1. Differences in *G* between the layer groups were negligible, so one value was used in the analytical solution for all three layer types.

### 2.5. Impact Testing and Residual Strength

The high-velocity impact was tested using a CZ Scorpion Evo 3 carbine (Česká zbrojovka a.s., Uherský Brod, Czech Repubic) shooting 9 mm × 19 mm Parabellum projectiles (Sellier & Bellot, Vlašim, Czech Republic) fired from a distance of 20 m. The projectile’s weight was 8 g, and it had a full-metal jacket and a round nose. The specimens were freely suspended inside two steel rope loops to eliminate the possible negative effects of boundary conditions. The shooter aimed into the centers of the specimens. Muzzle velocities were measured using a gun chronograph. The impact was monitored with two high-speed cameras.

Residual strength testing was performed in the same way as the aforementioned quasi-static testing, i.e., using the four-point bending experiment. Only the loading force was measured, as it was assumed that the dissimilar impact damage patterns would not yield consistent displacement and strain data.

## 3. Results and Discussion

### 3.1. Undamaged Specimens

**Figure 4:**
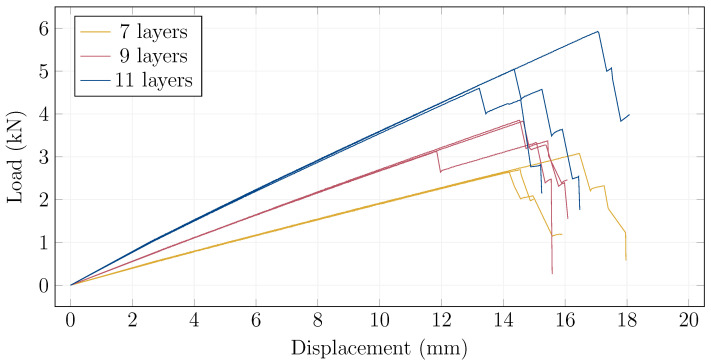
Load–displacement diagrams of all tested undamaged 7-, 9-, and 11-layer specimens (bottom, middle, top line groups, respectively).

The experimental results are summarized in Table 2, which shows the measured values at the points of the first glass ply cracks, which also corresponded to peak values, except for Specimen 9-2. Figure 4 shows the load–displacement diagrams of the bending experiments. The deflection values were averaged between the two center sensors, and offset by the average measurements of the sensors above the supports. The same layer groups had nearly identical behavior until the first glass ply cracks. However, the lowest-to-highest first glass ply cracking force relative differences are 14%, 18% and 22% for the 7, 9 and 11 layers, respectively. The stress values were calculated by multiplying the maximal values of strains (below load, strain gauge 5) by E=70 GPa.

Since the pre-cracking behavior in one layer group was similar, we could divide the deflection and strain values by the achieved first cracking forces and average the resulting values for each layer group. This is presented in the first part of Table 3. The second part shows the deflection and strains calculated using the analytical method, and the third part using the numerical simulations. Both were calculated to the highest forces from each layer groups, and the results were divided by the same force. Numbers in parentheses are the relative differences compared to the experiment.

Comparing the deflection values, there was good agreement with the experiment, as the relative differences did not exceed 10%. The analytical solution provides almost identical value for the lowest layer count while overestimating the deflection for the largest specimen. The trend of the numerical simulation’s results is the opposite in both aspects. The cross-sections of the specimens are symmetrical; therefore, the neutral axes lie in their centers and both the top and bottom strain absolute values must be the same in the analytical solution and the numerical simulation. The experimental results show higher strain values on the top surface by 2.8%, 2.0% and 1.2% for the 7, 9 and 11 layers, respectively, which are reasonably small differences, so the assumption holds. The experimental data could be influenced by manufacturing tolerances of the individual layers.

The second theoretical assumption is that the stress and, by extension, the strain on the top and bottom surfaces had a trapezoidal shape along the specimen’s length, like the bending moment of the four-point setup, with the constant moment region between loading points. This assumption was not met when analyzing the experimental data. The strain below the loading point was higher by 5.7%, 7.1% and 8.2% for the 7, 9, and 11 layers, respectively, compared to the center of the specimen. In addition, strain taken from gauges 4 and 7 (center of distance support-load) should theoretically be half of the strains measured in the theoretical constant moment region. However, it was 58%, 60%, and 61% lower than the central measurements for the 7, 9, and 11 layers, respectively. This caused a significant deviation in the analytical results for this point, even though the analytical model gave higher strain values, even for the other points, and especially for the 11-layer configuration. This, together with the rising deflection differences, could indicate that the EET method loses accuracy with higher layer counts.

In previous studies, the EET was compared to experiments with fewer layers where the agreement was good [25]. A comparison with a numerical simulation of a specimen with 5 identical glass layers also resulted in good agreement [23]. The numerical simulation in this case yielded similar strain values as those in the experiment with relative differences no higher than 10%, and accurately predicted the shape of the strain distribution along the specimen’s length. All of the experiments were carried out at an ambient temperature of 28°C. The performance of the glass, and the results of the analytical solution and numerical simulations are highly sensitive to the values of *G* of the interlayer. Therefore, care must be taken to accurately determine it, since the manufacturer’s specifications might be too general.

**Figure A1:**
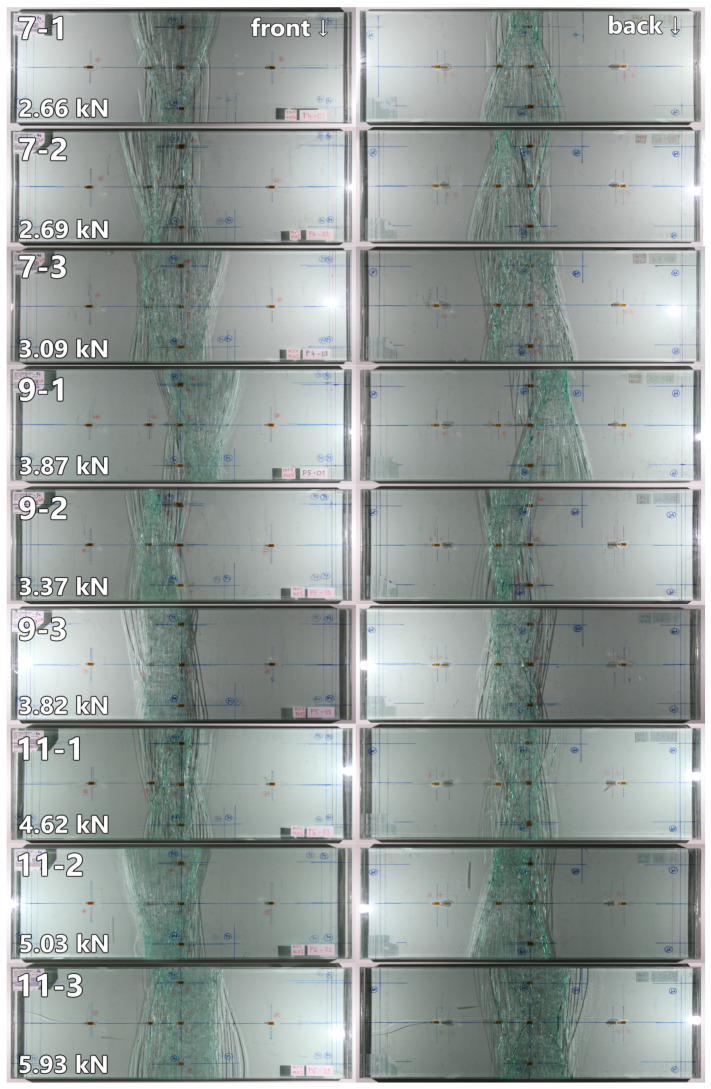
Specimens after quasi-static testing. Force values are peak forces.

European standard EN 572-1 [26] specifies the characteristic bending strength of float glass to be 45 MPa. The design then needs to apply reduction coefficients according to standard EN 16612 [27], which reduces this strength by 0.71 for 20 min (approximate duration of the experiments) loading time, and by an additional 0.8 for the damage initiation from the edge of the glass plate. This results in a 25.6 MPa bending strength. As seen in Table 2, all stress values were higher. This was an expected result, as the norm provides characteristic values. Differences in the measured values were likely caused by various pre-existing microcracks and the quality of the glass plates edges, which had a flat ground finish [28]. The final damage patterns are presented in Appendix A, Figure A1. The cracks were initiated at one of the edges and propagated in a fan-like pattern to the opposite side of one glass ply. The initial damage locations were not the same for each glass ply. The initiation point was always located between the loading points, although the fan patterns spread outside of it. Despite the strain values being the highest below the loading points, the initiation was not always present there. The size of the damaged area corresponded to the values of maximal tensile stresses. Specimen 11-3, which achieved 45.45 MPa stress, had the widest damage pattern without a clear single initiation point, but rather a wider damaged area on one side. On the other hand, Specimen 9-2, with 29.47 MPa, exhibited the smallest cracking area with a clear single point of initiation on all plies.

### 3.2. Projectile Impact Testing

**Figure 5:**
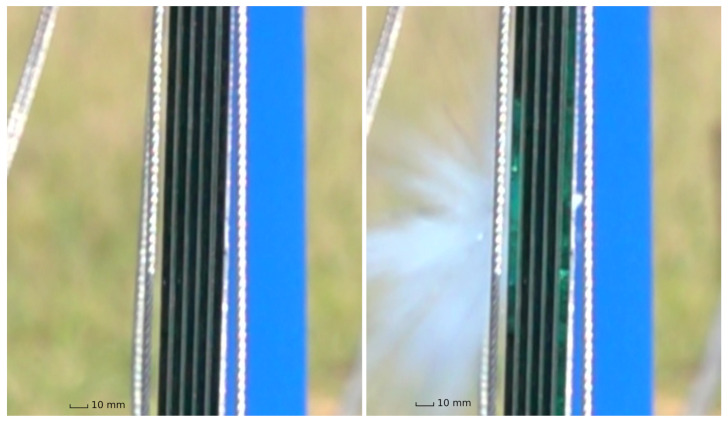
An example of high-speed camera frames from which glass damage was analyzed. (**Left**) before impact; (**right**) directly after impact. Impact direction from the left, both front and back plates damaged.

**Figure A2:**
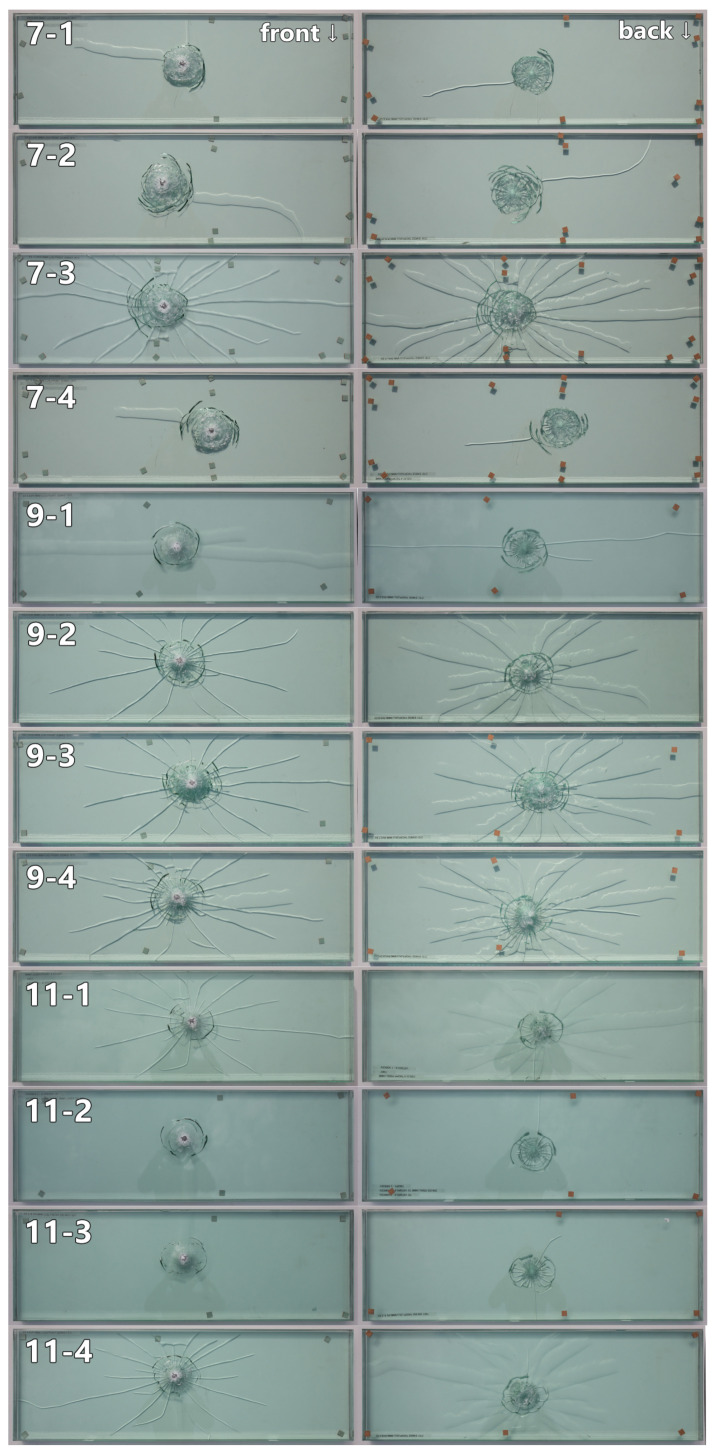
Specimens after impact testing.

After the ballistic testing had been conducted, the glass specimens were photographed and analyzed for damage with the help of the high-speed cameras. Comparing the frames of the high-speed camera before and after the impact from the side of the specimen clearly shows which glass layer developed cracks, since it changed how it reflected light in that angle. This is shown in Figure 5. All photographs of the tested specimens are in Appendix A Figure A2. As expected, the projectiles were stopped by the first glass layer, which is indicated by the flattened remains of the projectile stuck in a shallow crater (visible on the front faces in Figure A2). On the front glass plate, the crater contained a small area in the center with shattered glass. From this center, radial cracks propagated to a radius from approximately 150 mm up to 200 mm for the 7 and 9 layers, while the 11-layer specimens had a consistently smaller radius of around 140 mm. At the end of this damage area, several circular cracks were present. This front-plate damage was comparable among all specimens. Several specimens exhibited lone radial cracks propagating further than this outline, reaching the edge of the plate. The last glass plate, in cases where it was damaged, also exhibited a crater area from which a certain amount of material was ejected. Radial cracks were then present, reaching farther away from the crater compared to the front plate. On the other hand, the circular cracks were not apparent. The other glass layers were damaged only in Specimen 9-3. Table 4 shows a summary of the damaged layers for each specimen.

**Figure 6:**
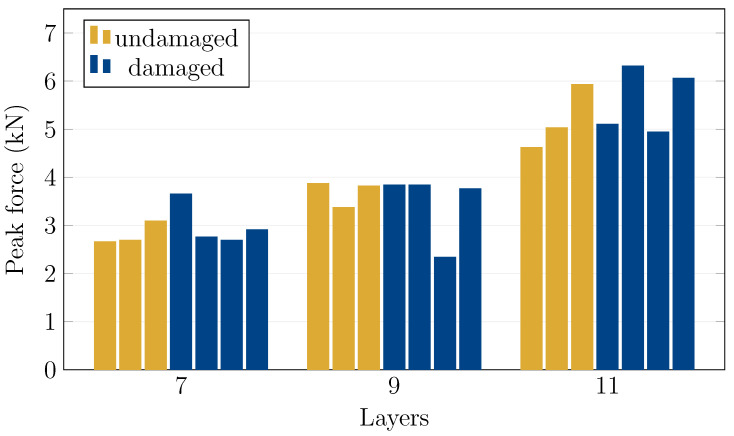
Comparison of peak forces between the undamaged and damaged specimens.

**Figure A3:**
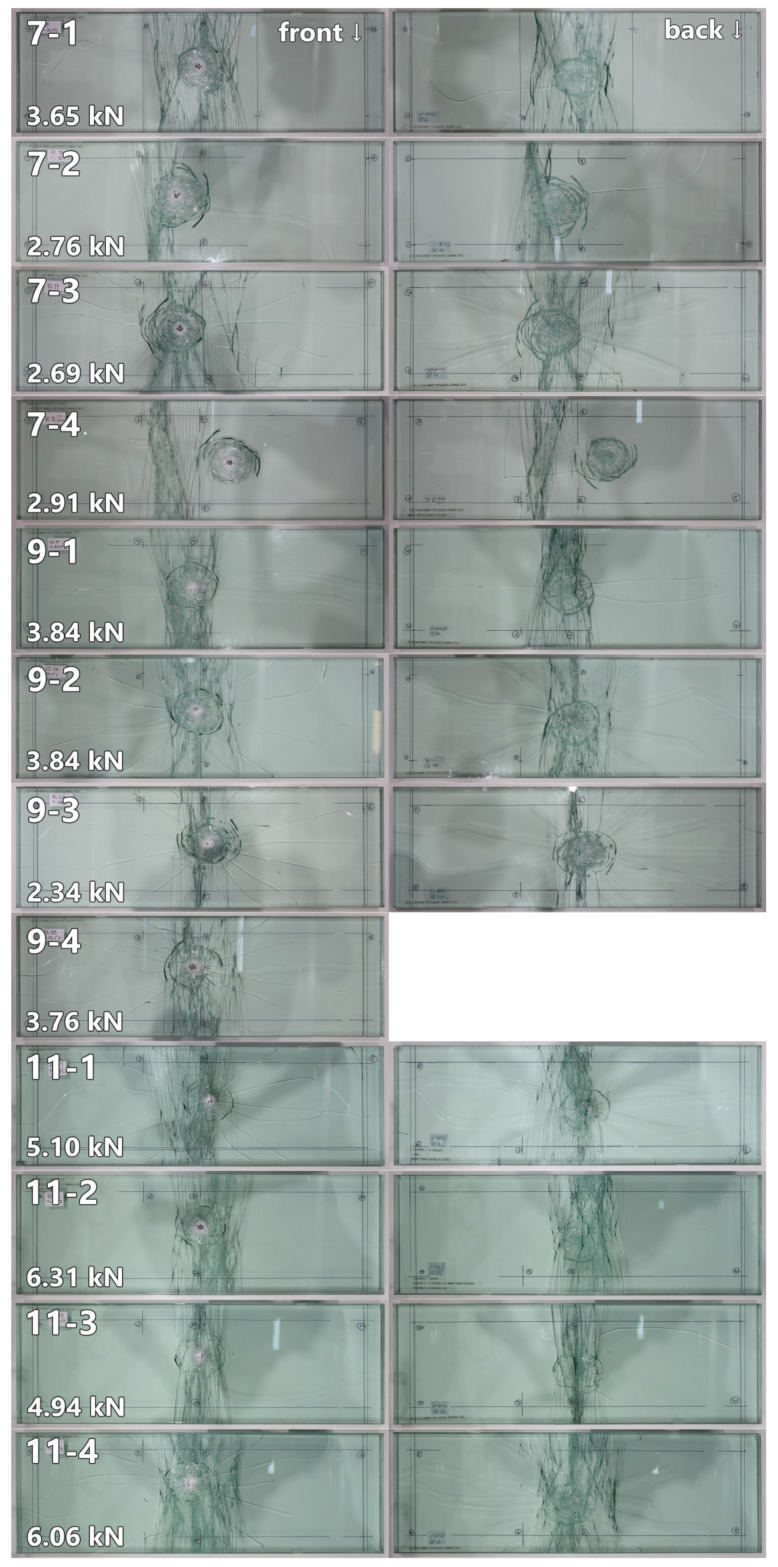
Specimens from the impact experiments after the subsequent quasi-static testing. Picture of the back of Specimen 9-4 is not available. Force values are peak quasi-static forces.

The damaged specimens were subjected to four-point bending tests, and the peak forces are presented in Table 4. Figure 6 shows a comparison of peak forces between the undamaged and damaged specimens. On average, this group of damaged specimens achieved 106%, 93% and 108% of the peak loads compared to the 7, 9, and 11 layers, respectively, of the undamaged group. However, this comparison should be understood as only roughly illustrative due to the higher spread of the values of both groups and statistically low amount of the specimens. Nevertheless, the ballistic damage had, in some cases, minimal influence on the resulting strength. Figure A3 in Appendix A shows the specimens after both the ballistic and the subsequent bending tests, and similarly to the undamaged specimens, the damage patterns correspond to the achieved peak forces.

Specimen 7-1 achieved the highest peak force for the 7-layer group, as the last layer was not damaged, and the final damage pattern was the largest of the group. On the other hand, the lowest peak force was achieved with Specimen 7-3, which had a damaged last layer with cracks reaching the edge of the ply, where the bending test cracking also initiated. Apart from Specimen 7-1, the cracking patterns had similar sizes, which corresponded to the similar peak forces. In the case of Specimen 7-4, the bending cracks completely bypassed the ballistic damage, but the cracking pattern was still relatively small, probably due to higher pre-existing microcracks on the edge. The location of the crater relative to the specimen’s center did not seem to correlate with the resulting mechanical performance.

The 9-layer group had the weakest, Specimen 9-3, since it had only the second to last layer undamaged with a very small final cracking pattern. The other specimens achieved similar performance despite Specimen 9-1 not being damaged on the last layer, although the sizes of the damage patterns were again similar.

The 11-layer group showed the lowest peak force for Specimen 11-3 despite its minimal ballistic damage, but the final damage pattern was the smallest, as expected. Specimens 11-1 and 11-4, despite having the last plies damaged, did not exhibit an initiation of the bending cracks according to the ballistic damage, as the radial ballistic cracks did not fully reach the edges of the last plies in the center of the span.

## 4. Conclusions

This study investigated the performance of laminated glass when subjected to high-velocity impact loading. The laminated glass was also analyzed using quasi-static bending experiments of two groups of specimens, undamaged or damaged by the impact. The specific conclusions can be summarized as follows:

- The EET analytical method was sufficiently accurate to predict the deflection and midspan strains. However, the relative differences compared to the experiments rose with the increasing number of layers. Deflections were higher by up to 8.7% and strains up to 16.8% in the center of the span.
- The presented numerical simulations could accurately predict the experimental results, including the strains and stresses with an error of up to 10%.
- All of the multilayer specimens were able to withstand the ballistic loading. The front plate was always damaged with a front crater. However, the back plate was not always damaged, regardless of layer count. Only in one case were more than just the front and back plates damaged.
- The residual post-impact bending performance was similar to that of the undamaged specimens, depending on the ballistic damage. The front damage played a negligible role. The back-plate damage needed to be extensive to influence the subsequent performance.
- Quasi-static cracks were always initiated from the edge of the specimen. The quality of the edges seemed to be more important than ballistic damage. In some cases, ballistic cracks reaching the edge of the specimen did not need to be the location of initiation of quasi-static cracking, in which case, the achieved strength was comparable to that of the undamaged specimens.
- In summary, the study showed that the ballistic damage did not significantly lower the post-crack bending performance. A structural element composed of laminated glass would be able to perform its function even after such an extreme loading event.

## Figures and Tables

**Table 1:** Parameters for the generalized Maxwell model with the long-term shear moduli $G_{\infty }=232.26$ kPa for TROSIFOL BG R20 (Trosifol, Germany; PVB-based foil) for $T_{0}=20$°C ($C_{1}=8.635$, $C_{2}=42.422$°C) from [24].

$\theta_{p}$ (s)	$G_{p}$ (kPa)
$10^{-5}$	1,782,124.2
$10^{-4}$	519,208.7
$10^{-3}$	546,176.8
$10^{-2}$	216,893.2
$10^{-1}$	13,618.3
$10^{0}$	4988.3
$10^{1}$	1663.8
$10^{2}$	587.2
$10^{3}$	258.0
$10^{4}$	63.8
$10^{5}$	168.4

**Table 2:** Results of the quasi-static bending experiments, undamaged specimens. Values for first glass ply cracking, which is equal to peak force points except for Specimen 9-2.

Specimen	Force	Deflection	Strain				Stress
			CB	CT	L	S	L
(Layers-#)	(kN)	(mm)	(µm/m)				(MPa)
7-1	2.66	14.18	451.6	—472.3	481.5	189.6	33.71
7-2	2.69	14.53	464.4	—470.9	487.3	190.5	34.11
7-3	3.09	16.46	527.6	—541.8	562.5	217.2	39.38
9-1	3.87	14.52	492.4	—490.7	529.0	194.4	37.03
9-2	3.12 ${}^{a}$	11.85	392.3	—410.4	421.0	162.8	29.47
9-3	3.82	14.65	485.6	—494.5	525.5	192.4	36.79
11-1	4.62	13.22	446.8	—456.1	485.0	175.2	33.95
11-2	5.03	14.34	456.8	—461.8	491.8	176.9	37.14
11-3	5.93	17.05	588.2	—591.1	649.3	230.0	45.45

CT—center top (average of strain gauges 1, 2, and 3, see Figure 2); CB—center bottom (6 and 8); L—below load (5); S—side (4 and 7); ^a^—peak force achieved for second glass ply crack at 3.37 kN.

**Table 3:** Comparison of the measured and simulated values. Average values for experiment calculated from Table 2. Numbers in parentheses are percentage differences compared to the experiment.

Layers	Deflection	Strain CB	Strain CT	Strain L	Strain S
	(mm/kN)	(µm/mkN)			
Experiment					
7	5.36	171.1	−176.1	181.5	70.8
9	3.79	126.6	−129.2	136.3	50.9
11	2.86	98.0	−99.2	106.7	38.2
Analytical solution					
7	5.35 (−0.1)	191.3 (11.8)	−191.3 (8.7)	191.3 (5.4)	95.6 (35.0)
9	3.94 (3.8)	140.8 (11.2)	−140.8 (9.0)	140.8 (3.3)	70.4 (38.3)
11	3.11 (8.7)	114.5 (16.8)	−114.5 (15.4)	114.5 (7.3)	57.3 (49.9)
Numerical simulation					
7	4.95 (−7.7)	184.8 (8.0)	−184.8 (5.0)	191.4 (5.5)	76.2 (7.6)
9	3.59 (−5.2)	135.3 (6.9)	−135.3 (4.7)	141.3 (3.6)	54.1 (6.3)
11	2.86 (−0.1)	107.6 (9.8)	−107.6 (8.4)	112.8 (5.7)	42.1 (9.6)

CT—center top (average of strain gauges 1, 2 and 3, see Figure 2); CB—center bottom (6 and 8); L—below load (5); S—side (4 and 7).

**Table 4:** Impact testing damage and subsequent quasi-static peak forces.

Specimen	Peak Force	Impact Damage	Muzzle Velocity
(Layers-#)	(kN)	(Glass Layers)	(m/s)
7-1	3.65	First	325
7-2	2.76	First	323
7-3	2.69	First, last	326
7-4	2.91	First	323
9-1	3.84	First	326
9-2	3.84	First, last	322
9-3	2.34	All but second to last	326
9-4	3.76	First, last	325
11-1	5.10	First, last	323
11-2	6.31	First	325
11-3	4.94	First	326
11-4	6.06	First, last	326

## Data Availability

Data are contained within the article.
